# Towards an Optimized Artificial Neural Network for Predicting Flow Stress of In718 Alloys at High Temperatures

**DOI:** 10.3390/ma16072663

**Published:** 2023-03-27

**Authors:** Chunbo Zhang, Qingyu Shi, Yihe Wang, Junnan Qiao, Tianxiang Tang, Jun Zhou, Wu Liang, Gaoqiang Chen

**Affiliations:** 1 Harbin Welding Institute Limited Company, Harbin 150028, China; 2 Department of Mechanical Engineering, Tsinghua University, Beijing 100084, China; 3 Smart Manufacturing Thrust, Systems Hub, Hong Kong University of Science and Technology (Guangzhou), Guangzhou 511466, China; ywang686@connect.hkust-gz.edu.cn

**Keywords:** artificial neural network, nickel alloys, accuracy, flow stress

## Abstract

Artificial neural networks (ANNs) have been an important approach for predicting the value of flow stress, which is dependent on temperature, strain, and strain rate. However, there is still a lack of sufficient knowledge regarding what structure of ANN should be used for predicting metal flow stress. In this paper, we train an ANN for predicting flow stress of In718 alloys at high temperatures using our experimental data, and the structure of the ANN is optimized by comparing the performance of four ANNs in predicting the flow stress of In718 alloy. It is found that, as the size of the ANN increases, the ability of the ANN to retrieve the flow stress results from a training dataset is significantly enhanced; however, the ability to predict the flow stress results absent from the training does not monotonically increase with the size of the ANN. It is concluded that the ANN with one hidden layer and four nodes possesses optimized performance for predicting the flow stress of In718 alloys in this study. The reason why there exists an optimized ANN size is discussed. When the ANN size is less than the optimized size, the prediction, especially the strain dependency, falls into underfitting and fails to predict the curve. When the ANN size is less than the optimized size, the predicted flow stress curves with the temperature, strain, and strain rate will contain non-physical fluctuations, thus reducing their prediction accuracy of extrapolation. For metals similar to the In718 alloy, ANNs with very few nodes in the hidden layer are preferred rather than the large ANNs with tens or hundreds of nodes in the hidden layers.

## 1. Introduction

In718 alloy is a precipitation-strengthened nickel alloy that possesses excellent properties, such as mechanical strength, strong oxidation resistance, and high corrosion resistance at temperatures up to 650 °C [1,2,3]. In718 alloy has been widely used in applications in which mechanical performance at high temperatures is important [2,4]. Certain manufacturing processes at high temperatures [5,6,7,8,9,10,11,12,13] of In718 alloy are generally involved to maintain its mechanical properties and the geometric part. In previous studies [14,15], finite element simulation was employed to predict and visualize the in-process thermal and mechanical variables during these manufacturing processes. With this information, the optimization of processing parameters could be expedited with more confidence, as both the thermal and mechanical variables are relevant to shape the final microstructure and thus the properties. An accurate model for predicting the flow stress at high temperatures is of critical importance to calculating the heat generation induced by material deformation and mechanical strain distribution. In this paper, we optimize an artificial neural network for quantitatively predicting the flow stress of In718 alloys at high temperatures for application in numerical simulations of manufacturing processes.

Quantitative models, such as the Sellars–Tegart model [16,17] and the Johnson–Cook model [18], have been proposed for predicting the flow stress of In718 alloy, which is dependent on temperature, strain, and strain rate. These models have been extensively employed in simulation models for manufacturing processes in which high processing temperatures are involved. For example, based on the hot compression experimental data of In718 superalloy, Jin et al. [19,20] established a constitutive equation based on the Sellars–Tegart model to predict the flow stress of In718 at high temperatures in their simulation of the forging processes. Wang et al. [21] also used the Sellars–Tegart model to predict the characterization of residual stresses and grain structures after hot forging of In718 alloy. Yang et al. [22] constructed a finite element model of the linear friction welding of In718 alloy using the Johnson–Cook material model and investigated the shearing and extrusion deformation types. The simulated results were validated through experiments. To provide in-depth understanding of the thermal-mechanical coupling behavior during linear friction welding, Geng et al. [23] developed a three-dimensional thermal mechanical model that included the strain-compensated Sellars–Tegart model for In718 based on the hot compression experimental data. Nan et al. [24] focused on constructing an accurate finite element model-based maximum entropy production principle to simulate the thermal-mechanical coupling process using DEFORM software, in which the Sellars–Tegart model of In718 superalloy was employed, and the simulation results were in good agreement with the experimental measurements.

In recent years, quantitative models based on artificial neural networks (ANNs) have been an important approach for determining high-temperature flow stress of metals and alloys with improved accuracy. Senthilkumar et al. [25] proved that ANNs have higher accuracy in predicting flow stress than the Sellars–Tegart model and the Johnson–Cook model. Sabokpa et al. [26] applied an ANN to predict the flow behavior of AZ81 magnesium alloy under high temperatures. To optimize the accuracy of ANNs, some researchers have adopted updated ANN algorithms called BP-ANN [27,28]. Huang et al. [29] further developed the GA-BP-ANN method, which also applied genetic algorithms (GAs), and precisely predicted the hot deformation of 40Mn steel. Wan et al. [30] introduced a particle swarm optimization (PSO) algorithm and developed a PSO-BP-ANN model to study the behavior of Zr-4 alloy with hot deformation.

Nevertheless, the accuracy of the models is highly dependent on the size and structure of the ANN. Moon et al. [31] investigated the prediction accuracy of an ANN with different sizes, and they also revealed that the hidden layer size and node number have independent influences on the accuracy. Sani et al. [32] predicted the constitutive equation of magnesium (Mg–Al–Ca) alloy by an ANN with one hidden layer and seven neurons per layer. Haghdadi et al. [33] established an ANN model to estimate the high temperature flow behavior of A356 aluminum alloy. The results indicated that an ANN with one hidden layer and 20 neurons per layer yielded the best trade-off between error and cost. However, there is still a lack of sufficient knowledge about what structure of ANN should be used for predicting metal flow stress.

In this paper, we train an ANN for predicting the flow stress of In718 alloys at high temperatures using our experimental data, and the structure of the ANN is optimized by comparing the performance of four ANNs in predicting the flow stress of In718 alloys at high temperatures. First, the experimental data, including the stress-strain value of In718 superalloy at high temperatures, are measured. Second, the measured data are used to train an ANN for the prediction of the stress-strain behavior. Third, the performance of ANNs with different hidden layers and numbers of neurons per layer is compared. Finally, an optimized ANN-based model for the prediction of high-temperature flow stress of In718 alloy is determined.

## 2. Materials and Methods

### 2.1. Hot Compression Tests

**Figure 1:**
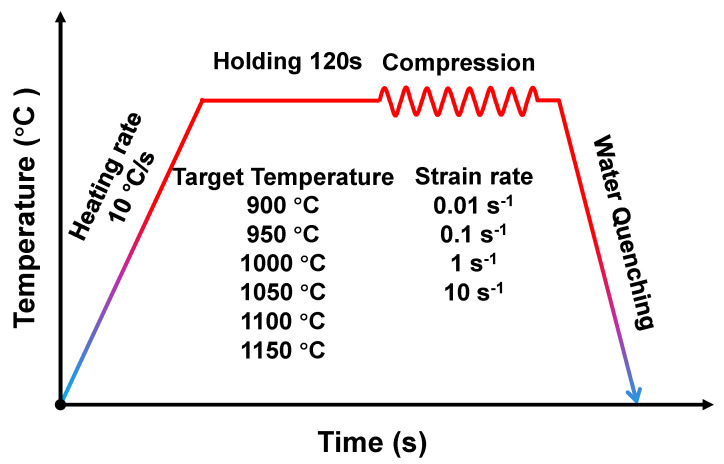
Schematic of thermomechanical history in the hot compression tests.

In this study, we used an In718 rod to test the flow stress. The chemical composition (wt. %) of In718 is given in Table 1. Before the tests, cylindrical specimens with a diameter of 8 mm and a height of 12 mm were prepared, and a K-type thermocouple was connected to the surface of the specimen for monitoring and controlling the temperature. In addition, uniaxial hot compression tests were performed using a Gleeble-3500 system at temperatures ranging from 900 °C to 1150 °C with an interval of 50 °C, and the experimental strain rates were 0.01/s, 0.1/s, 1/s, and 10/s, respectively. During hot compression, all specimens were heated to the target deformation temperature with a heating rate of 10 °C/s. Thereafter, the temperature was held at the targeted deformation temperature for 120 s to obtain a uniform microstructure [34]. Then, the specimens were compressed to a true strain of 0.6. In the compression tests, the friction between the end surfaces of the specimen and the anvils retarded the material flow, leading to a barrel-shaped specimen and consequent flow stress with a large measurement error. Therefore, graphite foils with a thickness of 0.05 mm were used as lubricants between the specimens and anvils to minimize the influence of friction during the hot compression tests. The detailed process is illustrated in Figure 1. After the hot compression tests, the specimen was quickly cooled to room temperature by water quenching. Consequently, the compressed specimens attained a near-net cylindrical shape with negligible bulging. As such, the calculation of the true stress-strain curve was based on assumption of uniform deformation. In the hot compression tests, loading force–stroke data were automatically recorded and were subsequently converted to stress–strain data according to the following equations:

(1) $$\sigma =\frac{FL}{\pi R_{0}{}^{2}\times L_{0}}$$

(2) $$\epsilon =ln\left(\frac{L}{L_{0}}\right)$$

where $L_{0}$ and $R_{0}$ are the original length and radius of the specimen, respectively; F is the loading force; and L is the instantaneous height of the specimen during compression.

### 2.2. Preparation for the Training Data

As described above, the temperatures in the experimental dataset were 900 °C, 950 °C, 1000 °C, 1050 °C, 1100 °C and 1150 °C, and the strain rates in the experimental dataset were 0.01/s, 0.1/s, 1.0/s, and 10.0/s per second. Under experimental loading, the strain varied from 0 to 0.6 with an interval of 0.05. As a result, there were in total 288 data points collected as training data. In each data point, the flow stress was collected as a result of the corresponding loading condition. This experimental dataset was applied in the ANN model to predict the flow stress. The performance of the ANN for predicting the flow stress of In718 alloy was evaluated in two aspects. One aspect is the ability to reproduce the flow stress at the datapoints included in the training data. The other aspect is the ability to reproduce the flow stress at datapoints other than the training data. Regarding the two aspects, the experimental data were divided into two datasets: the training dataset and the validating dataset. The training dataset and validating dataset are distinguished for regression and prediction purposes, respectively. Notably, the validating dataset does not engage in the training process. We randomly chose two sets of data from specific temperatures and strain rates. The training dataset included all the experimental data except the data at 950 °C/0.1/s and 1100 °C/1.0/s, and the validating dataset included the data at 950 °C/0.1/s and 1100 °C/1.0/s. The training dataset was noted as ‘dataset1’, and the validating dataset was noted as ‘dataset2’ for clarity of presentation. For the training process, we applied the ‘adam’ algorithm to perform the training. In future work, back propagation [35] methods could also be applied to increase the accuracy of training.

### 2.3. ANN Model for Predicting Flow Stress

**Figure 2:**
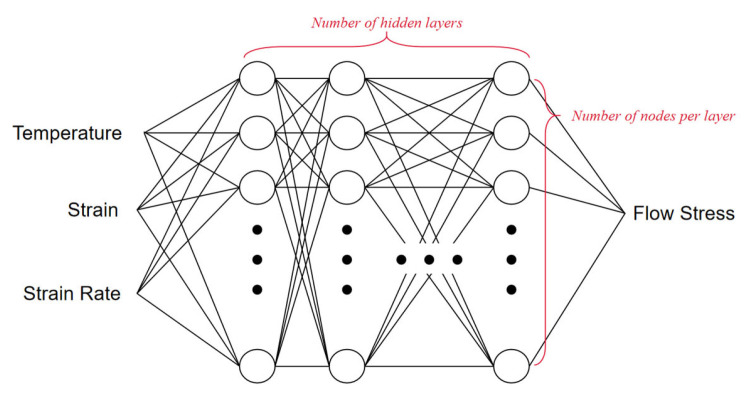
Illustration of the ANN for predicting the flow stress.

An ANN model was employed in this work for flow stress prediction. We illustrate the structure of the employed ANN model in Figure 2. It consisted of an input layer, hidden layers, and an output layer. In this model, the input layer consisted of three nodes, corresponding to three variables: temperature, strain rate, and strain. In terms of the output layer, it only consisted of one node, which is the value of flow stress. In the hidden layers, we applied to each hidden layer the same number of neurons, represented as N. The total number of hidden layers is represented as L. The structure of ANN is noted by N×L. In this work, we aimed to clarify the influence of ANN structure on accuracy. The ANNs with structures of 1×2, 1×4, 2×10, and 4×15 are taken as four typical cases in the analysis in this work.

For an ANN process, normalization of training data is generally used to ensure the efficiency and validity of training. According to the value range obtained from experiments, the input and output variables are normalized. The normalized temperature $T_{n}$ is defined by

(3) $$T_{n}=\frac{T-800}{450}$$

where T is the temperature in °C. The normalized strain rate ${\dot{\varepsilon }}_{n}$ is defined by,

(4) $${\dot{\varepsilon }}_{n}=\frac{{log}_{10}(\dot{\varepsilon })+3}{5}$$

where $\dot{\varepsilon }$ is the strain rate in 1/s. The normalized strain is taken as the true value of the measured strain, which is given by

(5) $$\varepsilon_{n}=\varepsilon$$

where ε is the measured strain. The normalized flow stress is given by,

(6) $$\sigma_{n}=\frac{\sigma_{FS}}{500}$$

where $\sigma_{FS}$ is the measured flow stress in MPa.

In this work, we used the scikit-learn tool [36] for training algorithms. The solver parameters are established to expedite the training process. ‘Adam’ solver was applied to improve the training result. We used a mode of constant learning rate of the solver, and the learning rate was set with a value of 0.001. The maximum iteration was set to be 1×10^8^, and the tolerance was set to be 1 × 10^−11^. The standard error is supposed to decrease at each iteration, while the training effect is weakened at each iteration. Thus, when the error reaches or values less than the tolerance, the training procedure will stop, and the solver will record the optimized ANN. If the error cannot provide a value greater than the tolerance, the solver will stop at the maximum iterations to save training time.

## 3. Results and Discussion

### 3.1. Measured and Predicted Flow Stress

**Figure 3:**
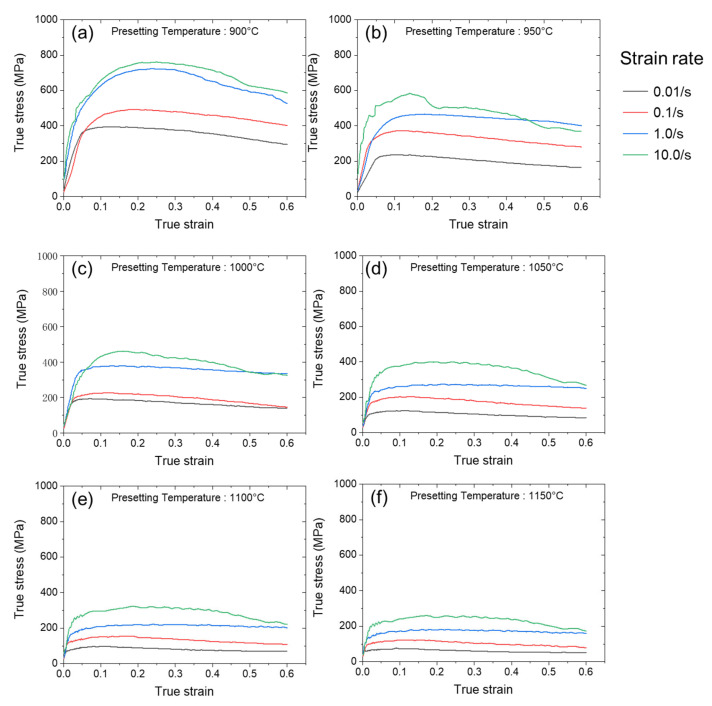
Experimentally measured stress-strain curves of In718 alloys at different temperatures and strain rates. The curves are grouped according to the testing temperature. The measured stress-strain are plotted in different colors for 0.01, 0.1, 1.0, and 10.0 in each figure. (**a**–**f**) are the experimental measured stress-strain curves at 900 °C, 950 °C, 1000 °C, 1050 °C, 1100 °C and 1150 °C.

Figure 3 shows the experimental measured stress-strain curves of the In718 alloy under the testing conditions. The measured strain-stress curves at 900 °C, 950 °C, 1000 °C, 1050 °C, 1100 °C and 1150 °C are plotted in Figure 3a–f, respectively. Each of the figures depicts the stress-strain curves at four strain rates at the corresponding temperatures. The relationship among flow stress and strain, strain rate, and temperature could be analyzed based on these measured curves. It could be found that the flow stress during the hot compression process in each condition changed in a similar pattern. When the sample was loaded from a zero-stress state, the stress first increased with the strain in a linear pattern in the elastic stage. Then, metal material yielded, and the plastic deformation began. In the plastic deformation state, the recorded stress value stabilized. As the material was loaded in a uniaxial state, the recorded compression stress equaled the required stress for deformation, i.e., the flow stress of the material. In this paper, we take the measured compression stress value in the plastic deformation state as the flow stress of the In718 alloy. It can be noted that the flow stress is related to the strain, strain rate, and temperature. The relationship between the flow stress of these thermomechanical variables is essential for modeling metal processing processes. In this paper, we used the measured flow stress data to train an ANN for predicting the flow stress of In718 alloy as a function of strain, strain rate, and temperature to develop a predictive model for the rotary friction welding process.

**Figure 4:**
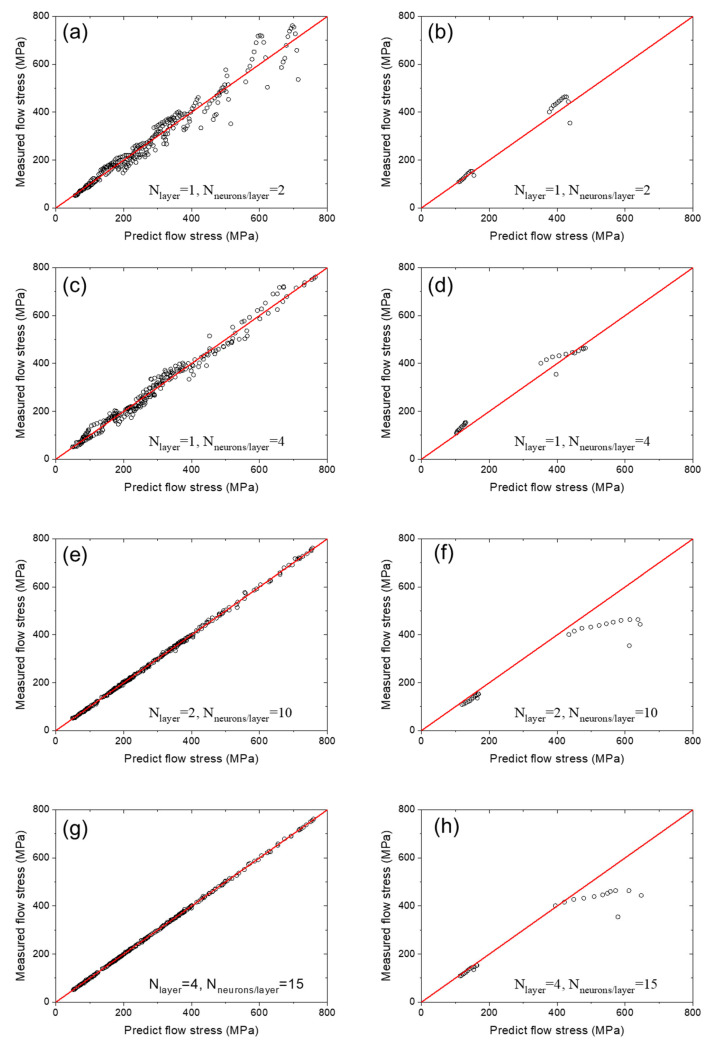
Plot of the measured flow stress versus the predicted flow stress of In718 alloy using ANNs with different structures. (**a**,**c**,**e**,**g**) are the training dataset, and (**b**,**d**,**f**,**h**) are the validating dataset. The ANN structure is noted inside each plot.

As described in Section 2.2, 264 data points in the training dataset (dataset1) were used for training the ANN, while 24 data points in validating dataset (dataset2) were used for validation. The result is visualized by the scattered plots in Figure 4. The measured flow stresses are plotted versus the predicted flow stress using an ANN with one layer and two neurons per layer for the training data ‘dataset1’ in Figure 4a and for the validating data ‘dataset2’ in Figure 4b. The measured flow stresses are plotted versus the predicted flow stress using an ANN with one layer and four neurons per layer for the training data ‘dataset1’ in Figure 4c and for the validating data ‘dataset2’ in Figure 4d. The measured flow stresses are plotted versus the predicted flow stress using an ANN with two layer and 10 neurons per layer for the training data ‘dataset1’ in Figure 4e and for the validating data ‘dataset2’ in Figure 4f. The measured flow stresses are plotted versus the predicted flow stress using an ANN with four layers and 15 neurons per layer for the training data ‘dataset1’ in Figure 4g and for the validating data ‘dataset2’ in Figure 4h.

The discrepancy between the experimental data and the predicted data is quantified using the root mean square error (RMSE) value, which is defined as

(7) $$RMSE=\sqrt{\frac{\sum_{i=1}^{N_{V}}{\left[\sigma_{predicted}\left(i\right)-\sigma_{exp}\left(i\right)\right]}^{2}}{N_{V}}}$$

where $N_{V}$ represents the number of data points in the corresponding dataset; $\sigma_{predicted}\left(i\right)$ and $\sigma_{exp}\left(i\right)$ represent the predicted flow stress by the ANN and the experimentally measured flow stress, respectively.

Table 2 shows the RMSE between the experimentally measured and predicted flow stress by the ANN for the training dataset and validating dataset for ANNs with different sizes. It could be found that the RMSE of the training dataset decreased as the size of the ANN increased. In contrast, the RMSE of the validating dataset reached its minimum when the ANN processed one hidden layer and four neurons per hidden layer. It is very interesting to note that increasing the size of the ANN from case 2 to case 3 or 4 resulted in a reduction in predicting accuracy. When the ANN had only one hidden layer and two nodes per hidden layer, the regression error was 34.96 MPa, and the prediction error was 30.30 MPa. When the ANN had four hidden layers and 15 nodes in each hidden layer, the RMSE of the training dataset was reduced to 2.06 MPa, while the RMSE of the validating dataset was as large as 81.52 MPa. It can be concluded that, with the increase in the size of the ANN, the ability of the ANN to retrieve the flow stress results from training sets is significantly enhanced; however, the ability to predict the flow stress results absent from the training does not monotonically increase with the size of the ANN but has an optimized performance when the ANN has one hidden layer and four nodes.

### 3.2. Predicted Strain Dependency of Flow Stress

**Figure 5:**
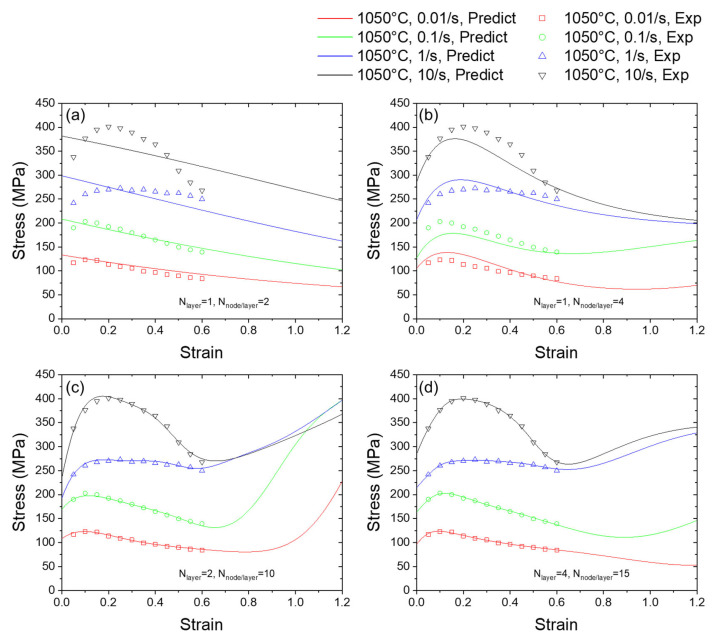
Predicted strain dependency of flow stress using ANNs with different structures. (**a**–**d**) are the predicted stress-strain curves by using ANNs with 1 × 2, 1 × 4, 2 × 10, 4 × 15 sizes.

As shown in Figure 5a–d, ANNs with different sizes provide different predictions of the strain dependency. When the ANN has one hidden layer and two nodes per hidden layer, the predicted flow stress decreases monotonically with the increase in the strain; obviously, the expected flow stress is nonlinear sets of values and cannot be well fitted with quasi-straight lines. Thus, the predicted strain dependency falls into underfitting and fails to predict the curve. After increasing the number of nodes in one hidden layer to four, the predicted curve is able to reproduce the strain hardening, where the strain is less than 0.2, and the strain softens with larger strains. As shown in Figure 5c,d, when the size of the ANN further increases, the discrepancy between the training points and predicted data is further reduced, indicating better fitting between the prediction data and the original training data. However, for the interval of data in which there are no training points, it could be found from Figure 5 that the predicted curves of flow stress versus strain also are prone to having more complicated shapes when the size of the employed ANN increases. It is worth noting that the curves have more fluctuation, the physical meaning of which might not be easily understood. For example, it can be noted in Figure 5c,d that, for strain larger than 0.6, the flow stress value suddenly bounces up and increases. In the practical manufacturing process, the sudden flow stress value increase is paradoxical to the commonsense of metal properties. To fully investigate this fluctuation behavior of the flow stress curve, we further conducted trials to predict the dependency on temperature and the strain rate of flow stress in the following contents.

### 3.3. Predicted Temperature Dependency of Flow Stress

**Figure 6:**
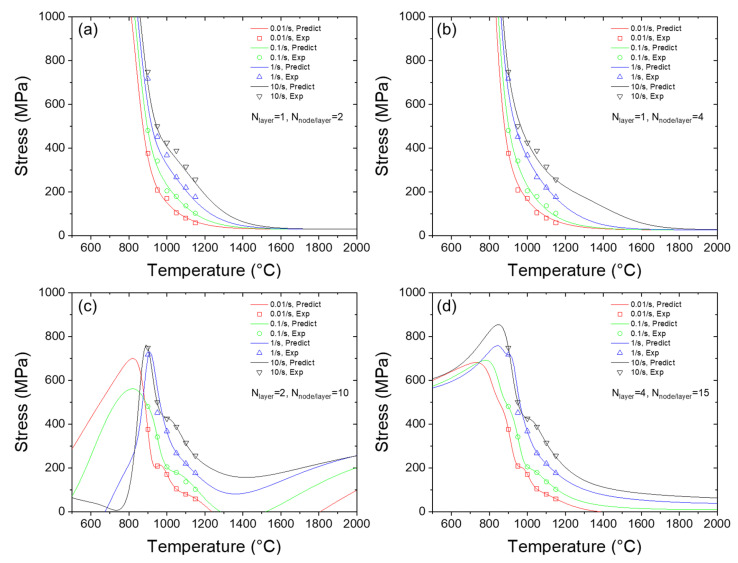
Predicted temperature dependency of flow stress using ANNs with different structures. (**a**–**d**) are the predicted stress-temperature curves by using ANNs with 1 × 2, 1 × 4, 2 × 10, 4 × 15 sizes.

Figure 6a–d plots the predicted flow stress versus the temperature using ANNs with different sizes. When the ANN has either one hidden layer/two nodes per hidden layer or one hidden layer/four nodes per hidden layer, the predicted flow stress decreases monotonically with the increase in the temperature. This outcome is consistent with the general law that the material is softened with the increase in temperature, which is also supported by the experimental data. However, as shown in Figure 6a,d, the predicted curve of flow stress exhibits more fluctuation, which makes the predicted curve not monotonical. For example, in Figure 6d, when temperature is less than 600 °C, the flow stress increases with the increase in temperature. Moreover, in Figure 6c the flow stress is prone to reaching a peak level in the temperature interval of 800–1000 °C and a valley level in the temperature interval of 1300–1400 °C, which cannot be explained by physical laws. It can also be noted that the abnormal behavior of the curve happens in the intervals outside of the training dataset’s temperature region. Thus, we deduce that it is due to the overfitting of the prediction curve by more complex ANNs. In other words, although it ‘seems’ that the fitting between the training datapoints and prediction datapoints is more accurate, the other predicted points are prone to being beyond the expected curve. It is also interesting to note that, when using small ANNs, there is no significant underfitting in temperature dependence.

### 3.4. Predicted Strain Rate Dependency of Flow Stress

**Figure 7:**
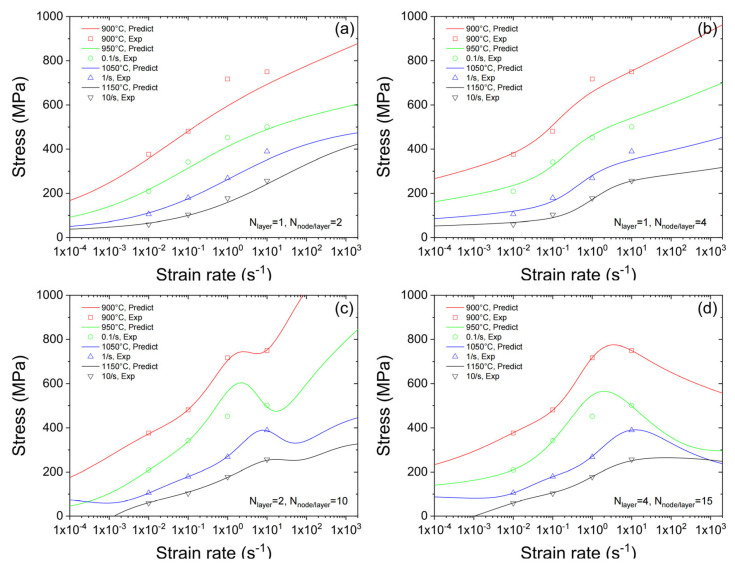
Predicted strain rate dependency of flow stress using ANNs with different structures. (**a**–**d**) are the predicted stress-strain rate curves demonstration of ANNs with 1 × 2, 1 × 4, 2 × 10, 4 × 15 sizes.

Figure 7a–d plots the predicted flow stress versus the strain rate using ANNs with different sizes. It could be found that the predicted flow stress increases monotonically with the increase in the temperature when the ANN has either one hidden layer/two nodes per hidden layer or one hidden layer/four nodes per hidden layer. This outcome is consistent with the general law that the material is hardened with the increase in temperature, which is also supported by the experimental data. However, as shown in Figure 7a,d, the predicted curve of flow stress exhibits more fluctuation, which makes the predicted curve not monotonic. It is also interesting to note that, when using small ANNs, there is no significant underfitting in strain rate dependence.

### 3.5. Predicted Flow Stress by Using the Optimized ANN

**Figure 8:**
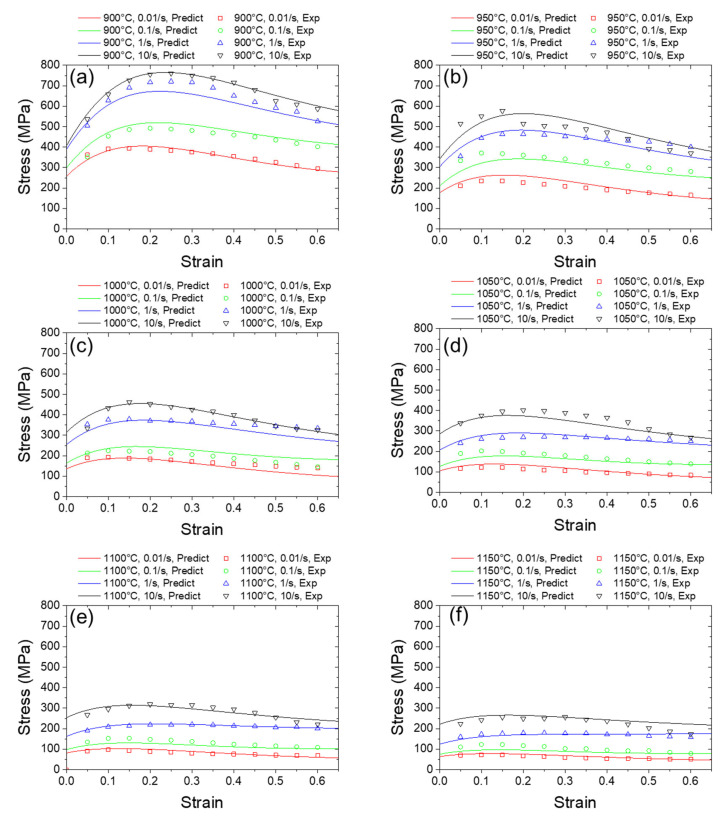
Predicted flow stress using the optimized ANN. (**a**–**f**) are the predicted stress-strain curves at 900 °C, 950 °C, 1000 °C, 1050 °C, 1100 °C and 1150 °C.

Consequently, by comparing the RMSE data and studying the prediction behavior of ANNs with different structures, it is found that ANNs with structural complexity of more than two layers and five nodes lead to an overfitting prediction result, hence reducing the prediction accuracy. Additionally, the time cost for training will increase with the increase in ANN size, while ANNs smaller than one layer and four nodes lead to a underfitting prediction result, which will reduce the accuracy as well. Thus, we choose the ANN with one hidden layer and four nodes per layer as an optimized structure. In other words, for In718 alloy and similar metals, ANNs with very few nodes in the hidden layer are preferred rather than large ANNs with tens or hundreds of nodes in the hidden layers. The predicted flow stress is plotted in Figure 8, grouped by strain rate and temperature. By choosing the ANN with one hidden layer and four nodes per layer, two desirable characteristics are demonstrated. First, the optimized ANN has good accuracy in predicting the flow stress. With limited experimental data training, the continuous and infinite case of predicted flow stress can be acquired. Second, the predicted stress-strain curves have good extensibility. For the interval during which the strain is less than the minimum experimental value or greater than the maximum experimental value, the flow stress values in the curves still fit the physical regulations of metal characteristics and avoid nonphysical fluctuations.

## 4. Conclusions

In this paper, an ANN is trained to predict the flow stress of In718 alloys at high temperatures based on experimental data. To optimize the structure of the ANN, the performance of four ANNs in predicting the flow stress of In718 alloy is comprehensively compared. The conclusions below are drawn:

(1) The comparison shows that the ability of the ANN to retrieve the flow stress results from training dataset is significantly enhanced as the size of the ANN increases, but the ability to predict the flow stress results absent from the training decreases when the ANN size exceeds a critical value.
(2) For In718 alloy and similar metals, ANNs with very few nodes in the hidden layer are preferred rather than large ANNs with tens or hundreds of nodes in the hidden layers. Specifically, the ANN with one hidden layer and four nodes possesses optimized performance for predicting the flow stress of In718 alloys in this study.
(3) The reason why there exists an optimized ANN size is discussed. When ANN size is less than the optimized size, the prediction, especially of the strain dependency, falls into underfitting and fails to predict the curve. When the ANN size is less than the optimized size, the predicted flow stress curves with temperature, strain, and strain rate will contain non-physical fluctuations, thus reducing its prediction accuracy for extrapolation.

The high-temperature flow stress of metal is a complex function of temperature, strain, and strain rate. Generally, ANN has the ability to predict high-temperature flow stresses of metals and alloys. This study shows that its ability is reliable unless the structure of the ANN is optimized by comprehensive research. However, it is obviously time-consuming to conduct the optimization whenever ANN is used to model the flow stress of metals. In future work, more effort should be made to develop a software toolbox to help engineers to use ANN for optimally predating the flow stress of metal.

## Figures and Tables

**Table 1:** Chemical Composition of In718 (Mass Fraction, %).

Element	C	Cr	Si	Cu	Mn	Mo	P	Ni	S	Pb
Mass Fraction, %	0.027	17.89	0.062	0.05	0.13	2.92	0.0084	53.14	<0.001	<0.0005
Element	Ag	Ti	Al	Nb	Ta	B	Bi	Co	Ca	O
Mass Fraction, %	<0.0005	1.01	0.51	5.42	0.003	0.038	<0.00003	0.28	<0.005	<0.0003
Element	Mg	Tl	Te	As	Se	Sn	N	Fe		
Mass Fraction, %	<0.003	<0.0001	<0.00005	<0.0025	<0.0003	0.0012	0.006	Bal		

**Table 2:** RMSE between the experimentally measured and ANN-predicted flow stress.

	RMSE between Measurement and Prediction	
	Training Dataset	Validating Dataset
Case 1	34.96 MPa	30.30 MPa
Case 2	21.21 MPa	23.43 MPa
Case 3	4.25 MPa	95.16 MPa
Case 4	2.03 MPa	81.52 MPa

## Data Availability

The data are available on request.
